# In Silico Analysis of Epitope-Based Vaccine Candidates against Hepatitis B Virus Polymerase Protein

**DOI:** 10.3390/v9050112

**Published:** 2017-05-16

**Authors:** Juzeng Zheng, Xianfan Lin, Xiuyan Wang, Liyu Zheng, Songsong Lan, Sisi Jin, Zhanfan Ou, Jinming Wu

**Affiliations:** 1 Department of Gastroenterology, The First Affiliated Hospital of Wenzhou Medical University, Wenzhou 325000, China; zhengjuzeng@sina.com (J.Z.); lynnxianfan@126.com (X.L.); 15158553395@163.com (L.Z.); jinsisi9907@126.com (S.J.); ouzhanfan12@126.com (Z.O.); 2 Department of Gastroenterology, Wenzhou People’s Hospital, Wenzhou 325000, China; wangxiuyan12@sina.com; 3 Department of Ultrasonography, The First Affiliated Hospital of Wenzhou Medical University, Wenzhou 325000, China; lanss_good@126.com

**Keywords:** bioinformatics, epitope, hepatitis B virus, polymerase, vaccine

## Abstract

Hepatitis B virus (HBV) infection has persisted as a major public health problem due to the lack of an effective treatment for those chronically infected. Therapeutic vaccination holds promise, and targeting HBV polymerase is pivotal for viral eradication. In this research, a computational approach was employed to predict suitable HBV polymerase targeting multi-peptides for vaccine candidate selection. We then performed in-depth computational analysis to evaluate the predicted epitopes’ immunogenicity, conservation, population coverage, and toxicity. Lastly, molecular docking and MHC-peptide complex stabilization assay were utilized to determine the binding energy and affinity of epitopes to the HLA-A0201 molecule. Criteria-based analysis provided four predicted epitopes, RVTGGVFLV, VSIPWTHKV, YMDDVVLGA and HLYSHPIIL. Assay results indicated the lowest binding energy and high affinity to the HLA-A0201 molecule for epitopes VSIPWTHKV and YMDDVVLGA and epitopes RVTGGVFLV and VSIPWTHKV, respectively. Regions 307 to 320 and 377 to 387 were considered to have the highest probability to be involved in B cell epitopes. The T cell and B cell epitopes identified in this study are promising targets for an epitope-focused, peptide-based HBV vaccine, and provide insight into HBV-induced immune response.

## 1. Introduction

HBV infection is a major public health problem with at least 250 million chronically infected individuals. More than 686,000 people die every year due to complications of hepatitis B that include cirrhosis and hepatocellular carcinoma [1]. Although safe and effective vaccines exist to prevent HBV infection, there is no cure for chronically infected individuals. The treatment of HBV infection includes conventional and pegylated interferon alfa, and five nucleos(t)ide analogues. Interferon has both antiviral and immunomodulatory activity, but its associated side effects limit clinical usage [2]. Nucleos(t)ide analogues target the DNA polymerase of HBV and inhibit reverse transcription of the pregenomic RNA into HBV DNA. However, the analogues have no effect on the elimination of covalently closed circular DNA (cccDNA) and often induce viral drug resistance, allowing relapse after treatment is stopped [3]. These shortcomings present an urgent need for an improved therapeutic method capable of HBV elimination or sustained suppression of viral load. Chronic HBV infection is characterized by impaired HBV-specific immune responses [4]. Restoring HBV-specific adaptive immunity will help to reduce antigen and viral load and ultimately achieve stable long-term control [5].

Research has shown that HBV hinders the liver’s innate immune responses, which are necessary in triggering adaptive immune responses [6]. Another factor contributing to the persistence of HBV infection is the existence of cccDNA [7]. Therefore, an optimal HBV therapy should be capable of not only stimulating innate and adaptive immune responses, but also of eliminating cccDNA.

HBV therapeutic vaccines aim to stimulate patients’ innate and adaptive immune response to effectively eliminate the virus. Poor clinical responses to therapeutic vaccines are probably due to the exhausted T cells not responding correctly to therapeutic vaccination [8]. The key to therapeutic vaccines is overcoming immune tolerance, and multiple studies have made progress in this direction. Examples include blocking programmed death (PD)-1/PD-L1 inhibitory signals on T cells in combination with therapeutic vaccination [9]. Some research has used immune adjuvant to improve the ability of therapeutic vaccination to overcome immune tolerance [10]. In addition, many studies have attempted to reduce the viral load by antiviral treatment to facilitate the induction of immune responses by therapeutic vaccination [11]. One study proved that HBsAg-pulsed autologous DCs had immune modulation capacity [12]. However, these strategies are effective in only a fraction of patients, and more effective immune-reconstitution treatment should consider multiple strategies simultaneously. Thus, additional strategies to overcome immune tolerance must be identified.

High HBV antigen levels, especially HBsAg, contribute to specific T cell exhaustion and limit the immunological response to therapeutic vaccination [13]. Persistent exposure of antigens to the immune system is responsible for immune tolerance, which is the reason for the poor clinical responses of therapeutic vaccines constituted by HBsAg and HBcAg. Theoretically, T cells only recognize a single viral epitope, and it is improbable that T cells would develop tolerance to all the proteins of a virus. HBV polymerase has low antigen levels in hepatocytes and few opportunities to contact the immune system. Therefore, it is unlikely that the immune system would develop tolerance to polymerase. A therapeutic vaccine constituted by polymerase epitopes might improve immunological responsiveness to therapeutic vaccination.

HBV DNA polymerase contains four domains and plays a vital part in HBV replication [14]. Therefore, inhibiting HBV polymerase would be an effective method for controlling HBV replication. Antigen levels would decrease once polymerase was inhibited, which would improve immunological responsiveness. Moreover, since steady-state plasma levels of cccDNA are dependent on viral replication, the inhibition of HBV polymerase will reduce cccDNA levels by means of its half-life [7]. For these reasons, we chose HBV polymerase as a promising antigen for a therapeutic vaccine.

Polymerase function is inhibited when nucleos(t)ide analogues incorporate into the elongating DNA chain and act as chain terminators, due to their lack of a 3’-OH, during HBV reverse transcription. Guanosine and adenosine analogs, such as entecavir and tenofovir, also impair protein priming [15]. HBV replication can be controlled by virus-specific CD8+ T cells through cytotoxic and non-cytotoxic methods [16], which implies that HBV-specific CD8+ T cells could target antigens inside the hepatocytes without lysis. The polymerase-specific CD8+ T cells stimulated by this vaccine will kill hepatocytes infected by HBV through direct lysis, which is mediated by perforin and granzyme. The polymerase-specific CD8+ T cells will also attack the polymerase in hepatocytes, non-cytotoxically, and will control virus replication, which will achieve the same effect as to nucleos(t)ide analogues without inducing drug resistance. Once the immune response targeting the HBV polymerase is stimulated, the effect will be permanent and may lead to sustained suppression of HBV replication.

In recent years, bioinformatics has garnered much scientific attention. Vaccines developed through bioinformatics are safer, more convenient, more efficacious, and less expensive than traditional vaccines [17]. Our present study was undertaken to select and analyze epitopes of HBV polymerase using a computerized approach and website assistance, after which molecular docking and MHC-peptide complex stabilization assay were utilized to determine the binding energy and affinity of epitopes to the HLA-A0201 molecule.

## 2. Materials and Methods

### 2.1. Amino Acid Sequence Retrieval

**Figure 1:**
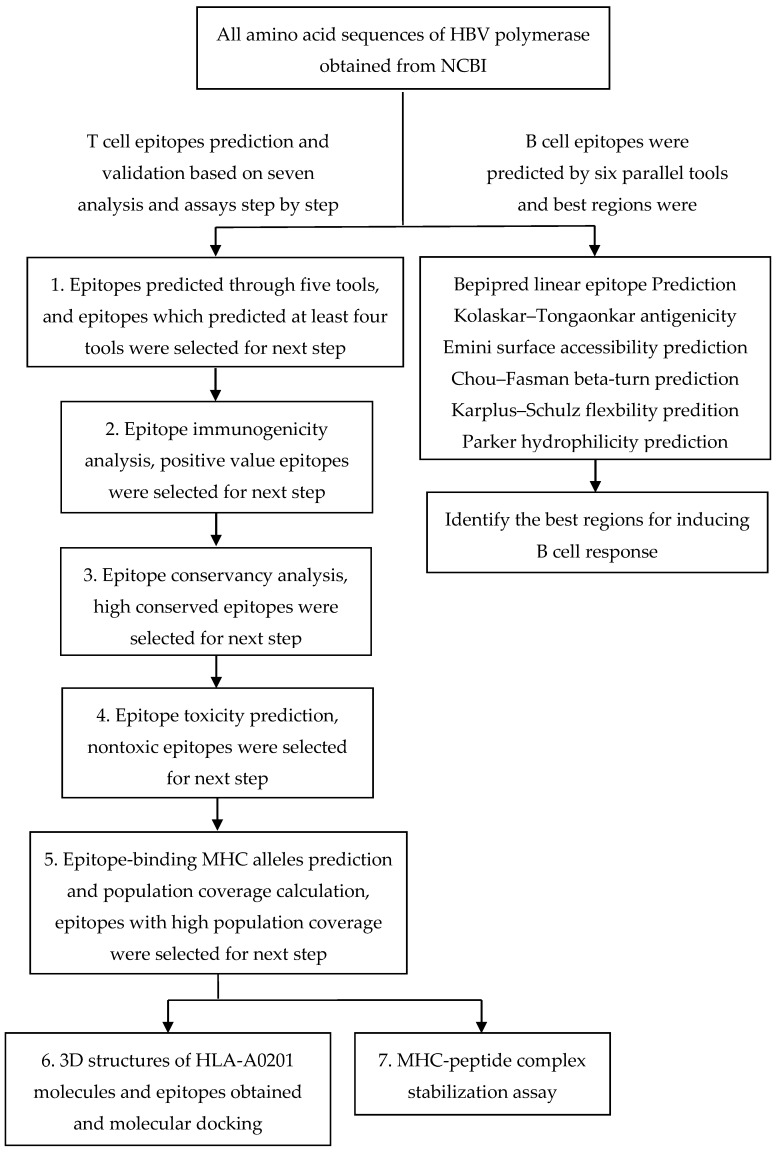
Graphical representation predicting T cell and B cell epitopes of HBV polymerase.

Different genotype protein sequences of HBV polymerase were obtained from GenBank of National Center for Biotechnology Information (NCBI). We selected complete sequences of all gene subtypes and excluded the sequences that constituted different genotypes. Sequences only containing partial sequences of genotypes were also excluded. Thirteen complete sequences, constituted by only one genotype, were selected (A, A1, A2, B, C, D, D3, D4, E, F2, F4, G, H). The lengths of these sequences ranged from 831aa to 845aa. (Accession of different genotypes, A genotype: BAN62840.1, A1 genotype: CCK33746.1, A2 genotype: CCK33754.1, B genotype: BAS53332.1, C genotype: BAQ95565.1, D genotype: CCH63720.1, D3 genotype: CCK33738.1, D4 genotype: CCK33750.1, E genotype: CCK33758.1, F2 genotype: CCK33698.1, F4 genotype: CCK33694.1, G genotype: BAD91284.1, H genotype: BAF49208.1). All sequences were stored in FASTA format. Since the B genotype is the most prominent genotype in China, the B genotype sequence was recorded for MHC-I binding, proteasomal C cleavage, TAP transport, immunogenicity, conservancy, toxin, population coverage, and B cell epitope analysis. A graphical representation predicting T cell and B cell epitopes is shown in Figure 1.

### 2.2. T Cell Epitope Prediction

To confirm whether the epitope could be presented on the surface of cells, the epitope prediction tools were used. Potential epitopes of HBV polymerase were predicted by T cell epitope prediction tools: IEDB MHC-I processing predictions, MHC-NP, netCTLpan1.1, RANKPEP, and netMHCpan3.0. Since HLA-A0201 was the most popular MHC allele and most MHC-I epitopes were nonapeptides, we selected the HLA-A0201 MHC allele and nonapeptide. Other parameters of each prediction tools were set to default.

The IEDB MHC-I processing predictions website aims to identify MHC-I ligands. This website provides approach of predicting peptides that are naturally processed from proteins and presented by MHC class I molecules. The MHC-I binding predictions were made on 12 June 2016 using the IEDB analysis resource consensus [18], which combines predictions from ANN aka NetMHC(3.4) [19,20], SMM [21], and Comblib [22] (http://tools.immuneepitope.org/processing/).

The MHC-NP server is designed for prediction of peptides naturally presented by the MHC molecules. This website employs data obtained from MHC elution experiments in order to assess the probability that a given peptide is naturally processed and binds to a given MHC molecule (http://tools.immuneepitope.org/mhcnp/) [23].

The netCTLpan1.1 server is designed for predicting cytotoxic lymphocyte (CTL) epitopes in protein sequences. This website integrates predicted peptide MHC-I binding, proteasomal C terminal cleavage performed using artificial neural networks (ANN), and TAP transport efficiency, which was predicted using weight matrix (http://www.cbs.dtu.dk/services/NetCTLpan/) [24].

RANKPEP server aims to predict epitopes of class I and class II MHC molecules based on protein sequences. This method predicts epitopes using position-specific scoring matrices (PSSMs) (http://imed.med.ucm.es/Tools/rankpep.html) [25,26,27].

NetMHCpan3.0 server is designed for predicting ability of peptide-MHC class I binding. This website predicts epitope binding to any MHC molecule using ANN (http://www.cbs.dtu.dk/services/NetMHCpan/) [28,29].

The top 50 predicted epitopes (about 6% of the total) were chosen, and epitopes which were predicted by at least four prediction methods were selected for further analysis.

### 2.3. Immunogenicity Prediction

Immunogenicity prediction tools was used to predict the ability of the epitope/MHC complex to elicit an immune response. The IEDB class I immunogenicity server is designed to predict the immunogenicity of a peptide MHC complex (http://tools.immuneepitope.org/immunogenicity/) [30]. Epitopes predicted using the method described above were then entered to evaluate their immunogenicity, and parameters were set to default. The epitope which obtained positive value was selected for conservation analysis.

### 2.4. Conservancy and Toxicity Analysis

In order to assess the conservancy level of epitopes within different genotype protein sequences, IEDB conservancy analysis was used. This analysis aims to calculate the degree of conservancy of epitopes within a given protein sequence (http://tools.immuneepitope.org/tools/conservancy/iedb_input) [31]. The sequence identity threshold was set to 100%, and other parameters were set to default.

To confirm the specific immune responses induced by epitopes that targets only the virus rather than the host tissue, the ToxinPred website was used to confirm epitopes’ nontoxicity (http://www.imtech.res.in/raghava/toxinpred/index.html) [32]. This website aims to predict epitope’ toxicity according to all the important physico-chemical properties. The parameters were set to default. The dataset used in this method included 1805 toxic peptides.

### 2.5. Population Coverage

HBV is a worldwide pandemic disease. To cover most chronic infected individuals, the IEDB population coverage method was carried out to estimate the rate of coverage of epitopes in regions with high HBV prevalence. Before estimating population coverage, the MHC-I alleles that would interact with epitopes were required. Therefore, epitopes were subjected to NetMHC4.0 server to predict the interacting MHC-I alleles. NetMHC4.0 server is designed for predicting ability of peptide-MHC class I binding and affinity between epitopes and MHC-I molecules using ANNs by detecting all MHC-I alleles. The MHC allele was set to HLA-A0201 and peptide length was set to nonapeptide. The threshold for strong binders and weak binders were respectively set to 0.5% and 2% rank (http://www.cbs.dtu.dk/services/NetMHC/) [19,33], and other parameters were set to default. The MHC alleles that bound to epitopes within the 2% rank were selected for further analysis. Because HBV is highly prevalent in East Asia, the Pacific nations, and sub-Saharan Africa [34,35], we selected East Asia, Southeast Asia, Central Africa, East Africa, South Africa, and West Africa to calculate the population coverage of epitopes using the IEDB population coverage tool. This website is designed for calculating epitopes’ population coverage of different regions based on the distribution of different MHC alleles to which epitopes bind. The calculation option was set to class I separate (http://tools.immuneepitope.org/tools/population/iedb_input) [36].

### 2.6. Molecular Docking

For the sake of ascertaining the binding affinities between the epitopes and MHC-I molecular structures, molecule docking was used. The epitope sequences were subjected to PEP-FOLD2.0 (http://bioserv.rpbs.univ-paris-diderot.fr/services/PEP-FOLD/) [37,38], a web-based server for three-dimensional (3D) conversion. This server aims at predicting peptide structures according to amino acid sequences, which was based on structural alphabet SA letter to describe the conformations of four consecutive residues, couples the predicted series of SA letters to a greedy algorithm and a coarse-grained force field. We obtained five PDB structures of each epitope. The HLA-A0201 was selected for docking and the molecular structure was downloaded from the RCSB Protein Data Bank (http://www.rcsb.org/pdb/home/home.do). The structure 4UQ3 of this database was the crystal structure of HLA-A0201 in complex with an azobenzene-containing peptide [39]. HLA-A0201 was simplified from this structure, and the water molecule was also removed. The docking analysis was performed by autodock4.2 [40]. The center grid box was set at X center = −0.156, Y center = 2.433, Z center = 19.959, and the number of points in each dimension was X = 60, Y = 40, Z = 58. We chose genetic algorithm (GA) and the number of GA runs was set to 100. The other parameters were set to default. The docking consequence was visualized for further interaction force analysis. The binding energy score was calculated, which was based on intermolecular energy, internal energy, and torsional energy.

### 2.7. Cell Line, Peptide Synthesis, and MHC-Peptide Complex Stabilization Assay

To determine the binding affinity of selected peptides to the HLA-A0201 molecule, we detected the upregulation of peptide-inducing HLA-A0201 molecules on T2 cells using flow cytometry. The transporter associated with the antigen processing (TAP)-deficient T2 cell line was purchased from American Type Culture Collection (ATCC) and maintained in complete RPMI-1640 medium. Candidate epitopes were synthesized and purified to greater than 99% purity (Shanghai Bootech BioScience & Technology Co. Ltd, Shanghai, China). The MHC-peptide complex stabilization assay was performed in accordance with the method described by Duan et al. [41], briefly, T2 cells (1 × 106/well) were incubated in 24-well plates with individual peptides (100 µM) for 18 h at 37 °C. The T2 cells without peptides served as background control. After incubation, T2 cells were incubated with fluorescein isothiocyanate (FITC)-conjugated anti-HLA-A2 mAb BB7.2, and analyzed using a BD AccuriC6 flow cytometer (Becton Dickinson, Mountain View, CA, USA). The mean fluorescence index (MFI) was recorded, which represented the fluorescence intensity of each well. Referred to other studies [41], we decided that the fluorescence indexes (FI) of each peptides were calculated as follows: FI = (MFI with individual peptides − background MFI)/(background MFI). Peptides with FI greater than 1 were regarded as high-affinity epitopes.

### 2.8. Identification of the B Cell Epitope

The goal of B cell epitope prediction was to determine the antigen recognized by B lymphocytes and initiate humoral immunity. To identify the B cell epitopes, IEDB B-cell epitope prediction was used. This website server is designed for prediction of B-cell epitope, which consisted of BepiPred linear epitope prediction [42,43,44], Chou–Fasman beta-turn prediction [45], Emini surface accessibility prediction [46], Karplus–Schulz flexibility prediction [47], Kolaskar–Tongaonkar antigenicity [48], and Parker hydrophilicity prediction [49].

## 3. Results

### 3.1. T Cell Epitope Prediction

In a preselected environment, the IEDB recommend, MHC-NP, netCTLpan1.1, RANKPEP and netMHCpan3.0 server predicted the potent epitopes from the HBV polymerase sequence. To improve the accuracy of epitope prediction, we selected epitopes which were predicted by at least four tools. In total, 30 epitopes were selected. Results are shown in Table 1.

### 3.2. Immunogenicity Prediction

Besides the epitope prediction, the binding affinity between peptide/MHC complex and TCR should be further analyzed. A high immunogenicity score was deemed to have high ability to stimulate naive T cells and induce cellular immunity. The epitopes selected using the above methods were subjected to IEDB immunogenicity prediction. The epitopes’ immunogenicity scores ranged from 0.22707 to −0.3485. Fifteen peptides with positive value scores were selected for further analysis. The results are depicted in Table 2.

### 3.3. Conservancy and Toxicity Prediction

To determine the conservancy status of selected peptides among the HBV polymerase proteins, multiple sequence alignments were carried out using IEDB conservancy analysis. Our analysis showed that two epitopes (RVTGGVFLV, WILRGTSFV) were 100% conserved in 12 other genotype sequences; epitope VSIPWTHKV scored 83.3%. Three epitopes (YMDDVVLGA, GLSRYVARL, HLYSHPIIL) showed 75% conservancy. Our data is shown in Table 2. The six epitopes that exceeded 75% conservancy in 12 other genotype sequences were selected for further analysis. The epitopes we selected showed conservancy approximating that seen in other research [50].

A qualified vaccine should have the ability to induce a specific immune response that targets only the virus rather than the host tissue. To confirm that inducement of cellular immunity by epitopes did not damage host tissue, we used a bioinformatics tool to predict the toxicity of epitopes; the data is depicted in Table 3. It is obvious that all six of the selected epitopes are nontoxic.

### 3.4. Population Coverage

**Figure 2:**
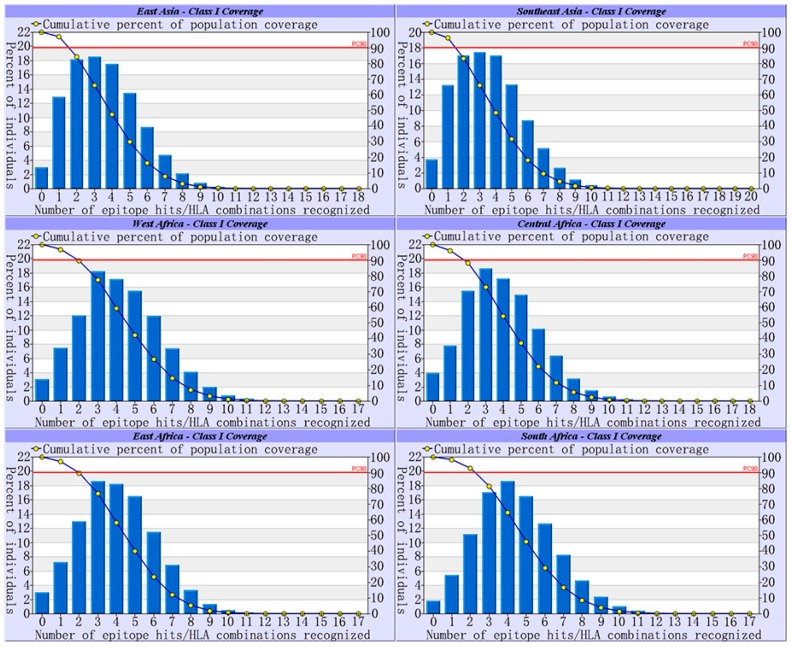
Population coverage of four selected epitopes in six regions with high HBV prevalence. Notes: In the graphs, PC90 represents the average number of epitope hits/HLA combinations recognized by 90% of the population.

To cover most chronic infected individuals, the IEDB population coverage method was used. The netMHC4.0 server was utilized to acquire MHC alleles with high epitope-binding force. The MHC alleles and binding ranks were shown in Table 3; population coverage in different areas is shown in Table 4 (minority MHC-I alleles were not found). The four epitopes RVTGGVFLV, VSIPWTHKV, YMDDVVLGA, and HLYSHPIIL, whose average population coverage exceed 50%, were subjected to molecular docking. The four epitopes indicated 97% coverage in East Asia, 96.26% coverage in Southeast Asia, 96.96% coverage in West Africa, 96.07% coverage in Central Africa, 97.02% coverage in East Africa, and 98.22% coverage in South Africa. The result is shown in Figure 2.

### 3.5. Molecular Docking and MHC-Peptide Complex Stabilization Assay

**Figure 3:**
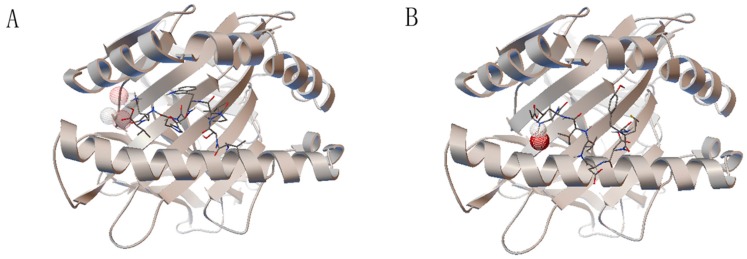
The molecule docking result. The HLA-A0201 molecule is shown by secondary structure and the epitopes bind to the groove between the α1 and α2 domains of the HLA-A0201 molecule. (**A**) The epitope VSIPWTHKV has two hydrogen bonds, to lysine 8 and lysine 146 of the HLA-A0201 molecule; (**B**) epitope YMDDVVLGA has only one hydrogen bond, to alanine 9 of the HLA-A0201 molecule. The hydrogen bonds were shown by wireframe.

**Figure 4:**
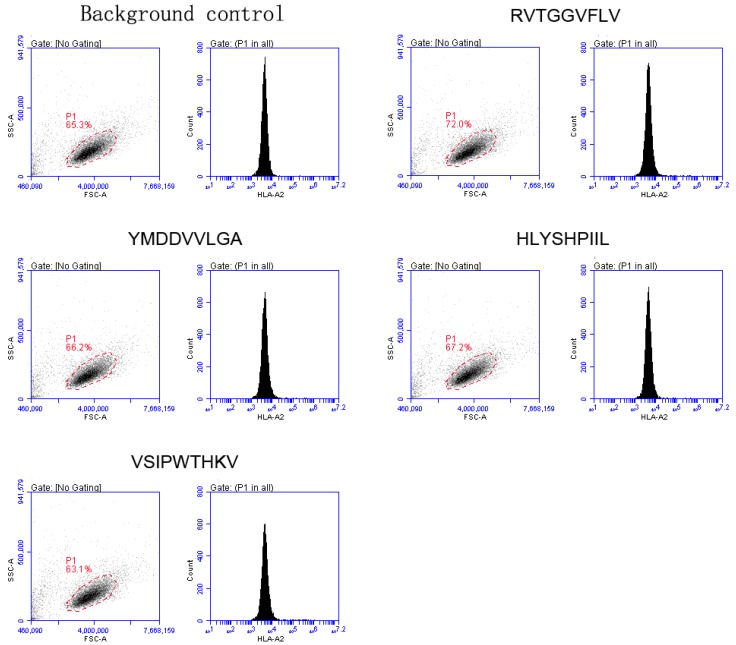
MHC-peptide complex stabilization assay. Flow cytometer result of background control and each epitope. The gate was sat on scatter plot, and the histogram is the fluorescence intensity of cells gated in scatter plot. The experiment was independently performed three times.

The epitopes selected by the above methods were validated by molecular docking. The binding energy between each structure and the HLA-A0201 molecule was calculated and ranked. Docking results are shown in Table 5 and Figure 3. Along with molecular docking, the selected epitopes were also validated by MHC-peptide complex stabilization assay. The results are depicted in Table 5 and Figure 4.

### 3.6. B-Cell Epitope Identification

Along with cellular immunity, humoral immunity is also required for viral elimination. B cell epitope identification was based on sequence characteristics of the antigen using amino acid scales, and different analysis methods were used to predict linear B cell epitopes.

**Figure 5:**
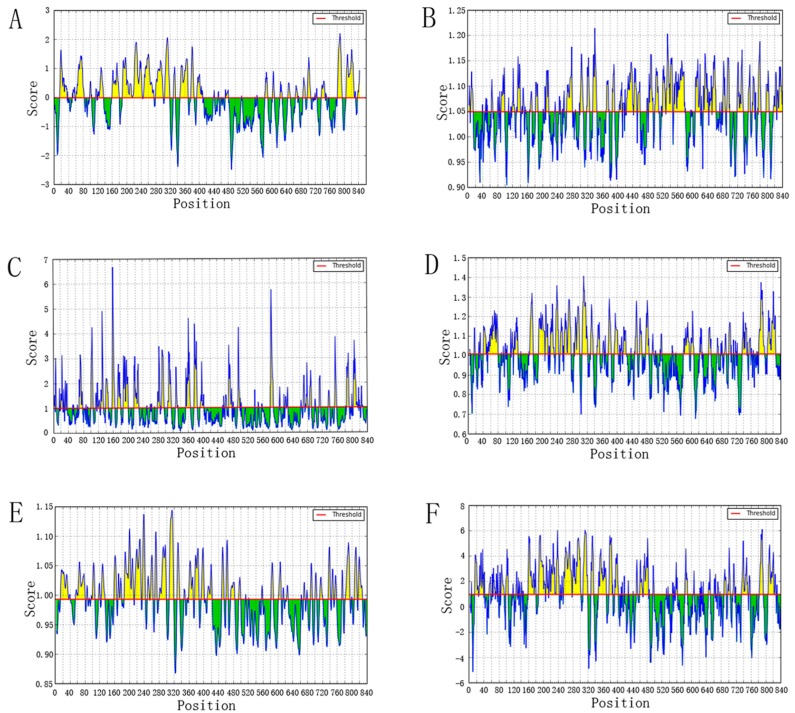
B cell epitopes prediction. The residues with scores above the threshold line are probably parts of epitopes, colored yellow. (**A**) BepiPred linear epitope prediction (threshold −0.026); (**B**) Kolaskar–Tongaonkar antigenicity (threshold 1.049); (**C**) Emini surface accessibility prediction (threshold 1.000); (**D**) Chou–Fasman beta-turn prediction (threshold 1.007); (**E**) Karplus–Schulz flexibility prediction (threshold 0.993); (**F**) Parker hydrophilicity prediction (threshold 0.952).

To predict B-cell epitopes, we should first identify the location of linear B-cell epitopes. Therefore, BepiPred linear epitope prediction was carried out using a hidden Markov model and propensity scale method. The result indicated that the average score is −0.026, with the minimum being −2.495 and the maximum, 2.197. The graph is shown in Figure 5A.

The Kolaskar–Tongaonkar antigenicity scale was used to predict antigenic determinants on proteins based on the physicochemical properties of amino acid residues. The frequency of occurrence in experimentally known segmental epitopes was also taken into consideration. The average antigenic propensity of the sequence was 1.049, with a minimum of 0.904 and maximum of 1.214. The highest score region was from 334 to 344 amino acid sequences. The result is shown in Figure 5B.

To be an epitope, surface accessibility was also taken into consideration. The Emini surface accessibility scale was carried out, with an average score of 1.000, a minimum of 0.065, and a maximum of 6.664. The highest surface-accessible region was between 158 and 165 amino acid sequences. The graph is shown in Figure 5C.

Experimental evidence has found that the beta turn and flexibility of proteins are also critical to B-cell epitope prediction. Hence, we implemented the Chou–Fasman beta turn prediction and the Karplus–Schulz flexibility scale to estimate the beta turn and flexibility of the proteins. The result of Chou–Fasman was an average score of 1.007, a minimum of 0.676, a maximum of 1.406, and the generated outcomes’ most recognized regions were from 308 to 316 amino acid sequences. The Karplus–Schulz result was an average score of 0.993, a minimum of 0.867, and a maximum of 1.144. The region between 307 and 320 amino acid sequences was considered the most favorable region in the flexibility prediction. The two graphs are shown in Figure 5D and Figure 5E respectively.

Finally, hydrophilicity should be analyzed. Hence the Parker hydrophilicity prediction was used to constructed a hydrophilic scale based on peptide retention times during high-performance liquid chromatography (HPLC) on a reversed-phase column. The result revealed an average score of 0.952, a minimum of −5.157, and a maximum of 6.100. The most hydrophilic region was from 785 to 794 amino acid sequences. The graph is shown in Figure 5F.

These six B cell epitope prediction tools divide sequences into different regions, and rank these regions based on probability of inducing B cell immune response. The top 2% of regions were selected to analyze the B cells’ epitope regions. We selected the regions predicted by at least four prediction tools for further analysis. We found that regions 307–320 and 377–387 contained overlapped regions predicted by at least four tools and were considered to have the highest probability to be involved in B cell epitopes. (Regions 307–320 included the BepiPred linear epitope predicted 313, 312, 314, 311, and 315 sites; the Chou–Fasman beta-turn predicted 308–316 region; the Karplus–Schulz flexibility prediction indicated 307–320 region; and the Parker hydrophilicity predicted 309–319 region. Regions 377–387 included the BepiPred linear epitope predicted 381 and 382 sites; the Emini surface predicted 377–387 region; the Chou–Fasman beta-turn predicted 377–383 region; and the Parker hydrophilicity predicted 377–387 region.)

## 4. Discussion

Our study predicted and selected four T cell epitopes, RVTGGVFLV, VSIPWTHKV, YMDDVVLGA, and HLYSHPIIL, on the basis of their immunogenicity, conservancy, toxicity, and population coverage. Epitopes VSIPWTHKV and YMDDVVLGA were found to have the lowest binding energy to the HLA-A0201 molecule, while epitopes RVTGGVFLV and VSIPWTHKV were found to have high affinity to the HLA-A0201 molecule. Regions 307–320 and 377–387 were considered to have the highest probability to be involved in B cell epitopes. Our research employed all criteria regarding epitope selection in an effort to obtain quality predictions. These four T cell epitopes and two regions involved in B cell epitopes will be validated by in vivo and in vitro experience in our subsequent research, and eventually we expect these epitopes will be found to constitute the basis of a HBV polymerase therapeutic vaccine.

There are many novel therapies for an HBV cure under investigation, including direct acting anti-virals (DAA) and host targeting agents (HTA). DAAs directly target the virus, and include entry inhibitors, polymerase inhibitors, site-specific cleavage of DNA agents, inhibitors of relax-circular DNA to cccDNA conversion, inhibitors of nucleocapsid assembly, capsid inhibitors, agents that knock down HBV RNA, viral proteins and HBV DNA, and agents that block HBsAg secretion. HTAs, which act by improving host immune ability, include therapeutic vaccines, immune checkpoint inhibitors, stimulators of exogenous interferon, and agents that induce APOBEC3A and APOBEC3B. These novel therapies are undergoing pre-clinical or clinical testing, and their effects should be further investigated [51,52].

There has been considerable development in the various types of therapeutic vaccines in recent years. The HBV surface-and-core-antigens-formulated vaccine Nasvac was proven to be able to stimulate both B and T cells in vitro [53]. An autophagosome-based HBV therapeutic vaccine was found to break HBV tolerance, suppress HBV replication and clear the HBV-infected hepatocytes in mouse models [54]. A nanoparticle-encapsulated unmethylated cytosine-phosphate-guanosine oligodeoxynucleotide from the HBV genome NP (HBV-CpG) was shown to induce robust IFN-α production and may act as an vaccine adjuvant [55]. Research demonstrates that GM-CSF may be useful as an immune adjuvant for both preventative and therapeutic purposes [56]. The fusion of DNA fragment encoding full-length HBsAg with cytotoxic-T-lymphocyte-associated antigen 4 (CTLA4) vaccine revealed high humoral and cellular immunity and the ability to suppress HBsAg and HBV DNA in both BALB/c mice and HBV transgenic mice [57]. The HBV polytope DNA vaccine, which used surface antigen epitopes, exhibited the ability to downregulate surface antigens and inhibit HBV DNA replication in an HBV transgenic mouse model [58]. An HBV DNA vaccine showed high levels of T cell and antibody response in both mice and human patients, and no adverse effects were observed [59]. However, the effects of HBV therapeutic vaccines in clinical use should be further determined. One clinical research study proved that HBV plasmid DNA (pSG2.HBs) vaccine was well tolerated but did not control HBV infection [60]. In chimpanzees chronically infected with a high viral HBV load, DNA encoding HBsAg+PreS2+HBcAg and DNA encoding IL-12 therapy failed to control HBV viremia [61].

In conclusion, numerous HBV therapeutic vaccines have been developed and subjected to clinical trials, including core antigen, e antigen, surface antigen, and recombined vaccines, but vaccines constructed only by polymerase have not been reported. Theoretically, the crucial role of polymerase in HBV replication and infection suggest that a polymerase antigen vaccine may be more effective than other vaccines [8]. Therapeutic vaccines have received significant development in recent years, and most of these are focused on how to induce a more powerful immune response, but less attention has been given to the immune response target. We believe that an excellent vaccine can not only induce a powerful immune response, but can also trigger an appropriate immune response, specifically inhibiting or eliminating the pathogen.

In recent years, bioinformatic analysis has been carried out as a significant approach to designing vaccines, including numerous methods and databases for designing subunit vaccines and epitope-based immunotherapy. Compared to the traditional approach, which administrates whole pathogens and raises several safety issues, the in silico tool is convenient and time saving [62]. Computational tools for T-cell epitope vaccine design could be divided into many steps. The binding to MHC-I [63], proteasomal C terminal cleavage [64] and TAP transport efficiency prediction [65] are based on protein sequences, and confirm whether the epitope can be presented on the surface of cells. Conservative prediction intends to provide broader protection for multiple genotypes and species [31]. Immunogenicity prediction is used to select the epitopes that are able to elicit T-cell response, and population coverage prediction is based on the distribution of MHC types in different regions [66]. Finally, B-cell epitope vaccine design focus on protein structures in order to predict the epitopes that can induce humoral immunity [67]. There are many B cell epitope prediction tools, such as IEDB antibody epitope prediction which we have introduced in part 3.6, Disco Tope 2.0 [68] and SEPPA [69]. The Disco Tope 2.0 server predicts B cell epitopes based on protein three dimensional structures. This tool calculates surface accessibility and a novel epitope propensity amino acid score, and the results are calculated by combining the contact numbers and the propensity scores of residues in spatial proximity. The SEPPA also predicts B cell epitope according to 3D protein structures. In a protein predicted by this tool, each residue will be given a score based on neighborhood residues’ information, and the higher score represent higher probability of the residue to be involved in an epitope. These tools give a better perspective of B cell epitope prediction and could be used to design vaccines.

The benefits of epitopes and vaccines designed by bioinformatics technology have been validated by appreciable research. A study in regard to glypican-1 (GPC3), which is a new prognostic factor for early hepatocellular carcinoma (HCC), showed that epitopes selected by technical websites have the ability to bind to T2 cells [70]. In another study, of PLAC1, T cell epitopes were predicted by SYFPEITHI, BIMAS, and NetCTL 1.2. In vitro assay indicated that CTLs induced by these epitopes from peripheral blood mononuclear cells (PBMCs) could release IFN-γ and lyse MCF-7 breast cancer cells. In vivo assay also proved that epitope p28 of PLAC1 could induce specific CTLs [71]. Moreover, epidermal growth factor receptor pathway substrate 8 (EPS8) is overexpressed in most human tumor tissue, while expressed to a lesser degree in normal tissue. The epitopes of EPS8 predicted by computer algorithms have been validated by T2 binding affinity assay and brefeldin-A decay assay. The CTLs induced by these epitopes were able to secrete IFN-γ and lyse EPS8-expressing cell lines [72]. Additionally, the epitopes of heparanase (Hpa) selected by bioinformatics technology could upregulate expression of the HLA-A2 molecule on T2 cells. The multi-epitope vaccines constructed by these epitopes were able to induce heparanase-specific CTLs to secrete IFN-γ and lyse tumor cells [73]. Overall, bioinformatics technology is a promising and rational approach for predicting and selecting epitopes.

It is crucial that epitopes identified through in silico analysis should be validated in vivo and/or in vitro experiments to certify their immunogenicity [74,75,76]. Although the nonapeptide epitopes analyzed in our study were determined by molecular docking and MHC-peptide complex stabilization assay, many experiments should be carried out to further validate the epitopes. In our forthcoming study, CTLs were generated from peripheral blood mononuclear cells (PBMCs) and HLA-A2.1/Kb transgenic mice with the stimulation of synthetic peptides. The next step is to evaluate the function of CTLs using enzyme-linked immunospot (ELISPOT) and cytotoxicity assay. The ability of inducing humoral immunity will be assessed to identify B cell epitope’ regions predicted in this study. Additionally, epitopes selected by the above methods could be subjected to construct DNA vaccine, the effects of which will be validated in further researches [77,78]. Besides the immunogenicity of epitopes, their capacity for inhibiting HBV replication also needs to be determined. The HepG2.2.15 cell is an HBV-producing human hepatocellular carcinoma cell, which can be used to measure the effects of an immunotherapeutic approach for HBV infection [79]. The inhibition of HBV replication should also be proved in vivo: HBV transgenic mice are an animal model that can be used to assess the effects of HBV therapeutic vaccines [13]. However, the epitopes predicted in the present research are binding to human MHC molecules; thus, the HBV polymerase epitopes targeting MHC molecules of HBV transgenic mice must be predicted and a corresponding vaccine should be constructed. Eventually, we hope to confirm that HBV polymerase vaccine is able to inhibit the HBV replication in vivo. 

## 5. Conclusions

Our research provides a novel and promising pathway to designing an HBV therapeutic vaccine. It is possible that a vaccine design based on this research will specially target HBV polymerase and inhibit HBV replication, which may achieve an effect comparable to nucleos(t)ide analogues, but without drug resistance. Furthermore, individuals with chronic HBV infections may be liberated from the inconvenience and financial burden of a lifelong medication regimen.

## Figures and Tables

**Table 1:** The selected epitopes were predicted by at least four tools.

Number	Sequence	IEDB Recommend (Total Score)	NetCTLpan1.1 (%Rank)	MHC-NP (Prob Score) ^1^	RANKPEP (Score)	NetMHCpan3.0 (Score)
1	FLLSLGIHL	1.23	0.3	0.703	74	0.8088
2	GLSRYVARL	−0.11	0.8	0.501	93	0.5567
3	HLYSHPIIL	0.24	1.5	0.3578	72	0.6134
4	GLYRPLLRL	−0.46	1.5	0.6303	81	0.555
5	FLLAQFTSA	−0.2	0.8	0.1685	67	0.8417
6	YMDDVVLGA	0.08	0.2	0.273	52	0.8052
7	RVTGGVFLV	−0.52	0.8	0.1529	76	0.6582
8	NLQSLTNLL	−0.74	2	0.2187	79	0.4628
9	LLDDEAGPL	−0.62	1.5	0.1576	53	0.515
10	NLGNLNVSI	−1.32	4	0.4817	80	0.4545
11	AVTHFLLSL	−0.65	3	0.1494	75	0.4462
12	RLGLYRPLL	−0.63	4	0.2311	50	0.483
13	SLYAAVTHF	−0.63	3	0.0983	81	0.393
14	SLYADSPSV	0.49	0.1	-	85	0.8179
15	ALMPLYACI	0.25	0.3	-	85	0.7411
16	LLSSDLSWL	0.59	0.4	-	80	0.6853
17	FLNKQYLNL	0.52	0.4	-	76	0.7243
18	LLAQFTSAI	−0.33	0.8	-	93	0.6915
19	NLYVSLMLL	0.02	1	-	93	0.6407
20	KLHLYSHPI	−0.39	0.8	-	59	0.6864
21	WILRGTSFV	−0.44	1.5	-	65	0.7009
22	HLPDRVHFA	−0.79	1.5	0.3226	-	0.5857
23	CLFHIVNLI	−0.53	1.5	-	75	0.5069
24	FTSAICSVV	−0.63	1	-	52	0.6207
25	SLNFMGYVI	−0.92	3	-	59	0.5115
26	NLIGTDNSV	−1.02	2	-	71	0.4586
27	HLLVGSSGL	−0.95	3	-	60	0.4516
28	FAVPNLQSL	−0.85	4	0.4014	-	0.3776
29	TLPQEHIVL	−1.18	5	0.2925	61	-
30	VSIPWTHKV	−1.45	3	0.2161	-	0.3956

^1^ The probability score predicted by MHC-NP represents the probability that the peptide is an epitope.

**Table 2:** Immunogenicity and conservancy prediction.

Number	Epitope ^1^	Immunogenicity Score	Conservancy (%)
1	CLFHIVNLI	0.22707	0.00
2	RVTGGVFLV	0.21088	100.00
3	HLPDRVHFA	0.20368	66.67
4	LLDDEAGPL	0.18597	41.67
5	VSIPWTHKV	0.18558	83.33
6	SLYAAVTHF	0.16679	0.00
7	TLPQEHIVL	0.14423	8.33
8	YMDDVVLGA	0.11902	75.00
9	GLSRYVARL	0.0969	75.00
10	GLYRPLLRL	0.05052	25.00
11	WILRGTSFV	0.0468	100.00
12	HLYSHPIIL	0.04347	75.00
13	AVTHFLLSL	0.04269	0.00
14	NLIGTDNSV	0.03386	8.33
15	RLGLYRPLL	0.02912	33.33

^1^ The epitopes were selected on the basis of immunogenicity score and conservancy.

**Table 3:** The seven epitopes with their interacting MHC-I alleles and toxicity.

Number	Sequence	Interacting MHC-I alleles and Binding Rank (%)	Toxicity
1	RVTGGVFLV	HLA-A*0201(0.60) ^1^,HLA-A*0202(1.60),HLA-A*0203(1.70),HLA-A*0205(1.80) HLA-A*0206(0.12),HLA-A*0207(2.00),HLA-A*0211(0.80),HLA-A*0212(1.30) HLA-A*0216(1.00),HLA-A*0219(0.30),HLA-A*0250(0.50),HLA-A*3001(1.90) HLA-A*3201(1.20),HLA-A*3207(0.20),HLA-A*6601(1.20),HLA-A*6802(1.40) HLA-A*6901(1.60),HLA-A*8001(0.80),HLA-B*1517(1.90),HLA-B*4013(0.40) HLA-B*4801(2.00),HLA-C*1203(1.10),HLA-C*1502(0.70)	Non-Toxin
2	VSIPWTHKV	HLA-A*0211(1.00),HLA-A*0212(1.50),HLA-A*0216(2.00),HLA-A*0217(2.00) HLA-A*0250(2.00),HLA-A*2301(1.80),HLA-A*3002(1.90),HLA-A*3207(0.70) HLA-A*3215(0.50),HLA-A*6601(0.50),HLA-A*6802(0.90),HLA-A*6823(0.90) HLA-A*6901(0.09),HLA-B*1517(0.80),HLA-B*5101(1.90),HLA-B*5701(1.40) HLA-B*5801(0.70),HLA-C*0602(1.30),HLA-C*0701(0.70),HLA-C*1203(0.20) HLA-C*1502(0.01)	Non-Toxin
3	YMDDVVLGA	HLA-A*0101(0.12),HLA-A*0201(0.02),HLA-A*0202(0.50),HLA-A*0203(0.30) HLA-A*0206(0.17),HLA-A*0207(0.25),HLA-A*0211(0.02),HLA-A*0212(0.05) HLA-A*0216(0.01),HLA-A*0219(0.06),HLA-A*0250(0.07),HLA-A*6901(0.80) HLA-B*0803(0.90),HLA-C*0401(1.70),HLA-C*0501(0.10),HLA-C*0802(1.60) HLA-C*1203(1.30)	Non-Toxin
4	GLSRYVARL	HLA-A*0201(1.00),HLA-A*0202(0.20),HLA-A*0203(0.50),HLA-A*0205(0.40) HLA-A*0211(1.10),HLA-A*0212(0.60),HLA-A*0216(1.00),HLA-A*0217(1.50) HLA-A*0219(0.80),HLA-A*0250(0.90),HLA-B*0802(0.40)	Non-Toxin
5	WILRGTSFV	HLA-A*0201(0.70),HLA-A*0203(0.50),HLA-A*0205(0.20),HLA-A*0206(0.30) HLA-A*0211(0.70),HLA-A*0212(0.50),HLA-A*0216(0.25),HLA-A*0217(1.20) HLA-A*0219(0.08),HLA-A*0250(0.30),HLA-A*3215(2.00),HLA-A*6802(1.40) HLA-A*6823(1.30),HLA-A*6901(0.07),HLA-A*8001(1.90),HLA-C*0401(0.90) HLA-C*0501(1.20),HLA-C*1203(1.70)	Non-Toxin
6	HLYSHPIIL	HLA-A*0201(0.80),HLA-A*0202(0.70),HLA-A*0203(0.40),HLA-A*0211(0.80) HLA-A*0212(1.30),HLA-A*0216(1.40),HLA-A*0217(1.30),HLA-A*0219(1.90) HLA-A*3201(1.00),HLA-A*3207(1.40),HLA-A*3215(1.10),HLA-A*6823(2.00) HLA-A*6901(1.40),HLA-B*0801(1.00),HLA-B*0802(0.80),HLA-B*0803(1.10) HLA-B*1502(0.80),HLA-B*1503(0.70),HLA-B*1509(0.09),HLA-B*3901(0.09) HLA-B*4013(0.17),HLA-B*4801(0.10),HLA-C*0303(0.80),HLA-C*0401(0.25) HLA-C*0602(0.80),HLA-C*0701(0.80),HLA-C*0702(0.12),HLA-C*1203(0.50) HLA-C*1402(1.50),HLA-C*1502(1.40),HLA-E*0101(1.10)	Non-Toxin

^1^ The scores in the brackets were the binding scores between epitopes and MHC-I molecules.

**Table 4:** Epitopes’ population coverage.

Number	Sequence	East Asia	Southeast Asia	West Africa	Central Africa	East Africa	South Africa	Average
1	RVTGGVFLV	49.22% ^1^	52.89%	57.67%	51.07%	62.62%	62.87%	56.05%
2	VSIPWTHKV	35.60%	34.45%	73.47%	73.59%	76.95%	86.35%	63.40%
3	YMDDVVLGA	50.96%	52.64%	65.85%	58.72%	56.68%	36.45%	53.55%
4	GLSRYVARL	24.26%	25.49%	32.03%	34.10%	32.15%	25.07%	28.85%
5	WILRGTSFV	44.40%	39.88%	59.94%	50.82%	55.19%	40.40%	48.44%
6	HLYSHPIIL	94.14%	93.16%	85.10%	85.40%	82.18%	91.75%	88.62%

^1^ The data was shown by the percentage of population the epitopes covered.

**Table 5:** Binding energy and fluorescence index.

Number	Epitopes	Binding Energy	Fis ^1^
1	RVTGGVFLV	0.28	1.09
2	VSIPWTHKV	−5.33	1.50
3	YMDDVVLGA	−3.26	0.53
4	HLYSHPIIL	3.75	0.64

^1^ Fluorescence index (FI) was calculated as described in Section 2, Materials and Methods.
